# Laparoscopic Proximal Gastrectomy *Versus* Laparoscopic Total Gastrectomy for Proximal Gastric Cancer: A Systematic Review and Meta−Analysis

**DOI:** 10.3389/fonc.2020.607922

**Published:** 2021-01-21

**Authors:** Peirong Tian, Yang Liu, Shibo Bian, Mengyi Li, Meng Zhang, Jia Liu, Lan Jin, Peng Zhang, Zhongtao Zhang

**Affiliations:** Department of General Surgery, Beijing Friendship Hospital, Capital Medical University and National Clinical Research Center for Digestive Diseases, Beijing, China

**Keywords:** laparoscopic total gastrectomy, laparoscopic proximal gastrectomy, proximal gastric cancer, meta-analysis, systematic review

## Abstract

**Background:**

To compare laparoscopic proximal gastrectomy (LPG) and laparoscopic total gastrectomy (LTG) with regard to outcomes, including efficacy and safety, in patients with proximal gastric cancer.

**Methods:**

Original English-language articles comparing LPG and LTG for proximal gastric cancer up to November 2019 were systematically searched in the Embase, PubMed, Cochrane Library, Web of Knowledge, and ClinicalTrials.gov databases by two independent reviewers. Our main endpoints were surgery-related features (operation time, blood loss, harvested lymph nodes, and postoperative hospital stay), postoperative complications (anastomotic leakage, anastomotic bleeding, anastomotic stenosis, and reflux esophagitis), and oncologic outcomes (5-year overall survival and recurrent cancer).

**Results:**

Fourteen studies including a total of 1,282 cases (510 LPG and 772 LTG) were enrolled. Fewer lymph nodes were harvested (WMD = −13.33, 95% CI: −15.66 to −11.00, *P* < 0.00001) and more postoperative anastomotic stenosis (OR = 2.03, 95% CI: 1.21 to 3.39, *P* = 0.007) observed in LPG than LTG. There were no significant differences in other explored parameters between the two methods. However, based on a subgroup analysis of digestive tract reconstruction, LPG with esophagogastrostomy (LPG-EG) had shorter operative time (WMD = −42.51, 95% CI: −58.99 to −26.03, *P* < 0.00001), less intraoperative blood loss (WMD = −79.52, 95% CI: −116.63 to −42.41, *P* < 0.0001), and more reflux esophagitis (OR = 3.92, 95% CI: 1.56 to 9.83, *P* = 0.004) than was observed for LTG. There was no difference between LPG performed with the double tract anastomosis/double-flap technique (DT/DFT) and LTG.

**Conclusion:**

LPG can be performed as an alternative to LTG for proximal gastric cancer, especially LPG-DT/DFT, with comparable safety and efficacy.

## Introduction

Gastric cancer remains the fifth most frequently diagnosed cancer and the third most common cause of cancer-related death worldwide, especially in Eastern Asia, Eastern Europe, and South America (1, 2). The epidemiological characteristics of gastric cancer have changed over the last several decades. Although the overall incidence of gastric cancer has decreased, the incidence rate of proximal gastric cancer has been increasing in both Western and Asian countries (3, 4). Despite developments in multimodal therapy strategies, such as chemotherapy, radiotherapy, targeted therapy, and immunotherapy, the most effective therapy for potentially curable proximal gastric cancer has remained surgical resection (5). With the rapid development of minimally invasive surgery, clinical applications involving laparoscopic gastrectomy have been widely accepted as standard surgeries for proximal gastric cancer (6, 7). There are two main types of laparoscopic surgical strategies: laparoscopic total gastrectomy (LTG) and laparoscopic proximal gastrectomy (LPG).

Compared to LPG, LTG can achieve a longer tumor-free distal resection margin and more radical lymphadenectomy, which seem to have better curative effects. More significantly, compared with LPG, LTG has few postoperative complications (8). However, in many retrospective studies, compared to LPG, LTG has shown only slightly superior results in this poor-outcome cancer (9–13). Additionally, anemia, weight loss, and symptoms, such as heart burn, nausea, and vomiting (known as postgastrectomy syndrome), are frequent postoperative complications in LTG patients and must be considered (14–17). In contrast, LPG has theoretical advantages in terms of postoperative nutritional status and anemia because the gastric reservoir is preserved and gastric acid secretion and intrinsic factors are maintained (18–22). However, an unavoidable consequence of traditional LPG-EG is reflux esophagitis, which is the main factor that reduces postoperative quality of life in this population. Thus, the adequate surgical technique for the treatment of proximal gastric cancer remains controversial. While the feasibility and safety of these two methods have been demonstrated in a variety of studies, most of them were single-center studies with small sample sizes and limited follow-up periods. Previous meta-analysis relied on data mostly obtained from conventional open surgery (23–27). To overcome these limitations, a meta-analysis of studies comparing LPG and LTG for proximal gastric cancer should be performed. Therefore, we conducted this systematic review and meta-analysis to systematically review the surgery-related features, postoperative complications and oncologic outcomes of LPG and LTG for proximal gastric cancer.

## Methods

This systematic review was performed according to the PRISMA statement.

### Literature Search

The literature published in English from the inception dates of each of the following electronic databases up to November 2019 was searched: PubMed, Embase, Cochrane Library, Web of Knowledge, and ClinicalTrials.gov. The keywords “laparoscopic,” “proximal gastrectomy,” “total gastrectomy,” and “proximal gastric cancer” were used. Search strings of PubMed were as follows: ((((((((((stomach neoplasm[MeSH terms])) OR (cancer of stomach)) OR (cancer of the stomach)) OR (gastric cancer)) OR (gastric neoplasms)) OR (neoplasms, gastric)) OR (neoplasms, stomach)) OR (stomach cancer)) AND (((((((((((laparoscopy[MeSH terms]) OR (celioscopy)) OR (laparoscopic assisted surgery)) OR (laparoscopic surgery)) OR (laparoscopic surgical procedure)) OR (peritoneoscopy)) OR (procedure, laparoscopic surgical)) OR (procedures, laparoscopic surgical)) OR (surgery, laparoscopic)) OR (surgical procedure, laparoscopic)) OR (surgical procedures, laparoscopic))) AND ((total gastrectomy[title/abstract]) AND (proximal gastrectomy[title/abstract])). All titles, abstracts and related citations were scanned and reviewed. Reference lists from primary studies and review articles were also examined manually to search for additional publications. Two authors individually conducted the literature search and crosschecked their search results.

### Study Selection

Duplicate search results were first excluded. Studies were included in this research according to the following criteria: 1) clinical studies that compared LPG with LTG for proximal gastric cancer, and 2) LPG/LTG that was performed with either a laparoscopy-assisted or total laparoscopic approach.

Papers meeting any of the following criteria were excluded: 1) studies including other types of gastric resection, unless the data were presented separately; 2) conference abstracts, reviews, comments, case reports, noncomparative studies, nonrelevant topic papers, non-English papers, and animal studies; and 3) duplicated publications or publications that did not provide sufficient data.

### Quality Assessment of the Studies

Since all the included studies were nonrandomized, the Newcastle-Ottawa Scale (NOS) was used to judge study quality, as recommended by the Cochrane Collaboration. The maximum score achievable on the NOS is nine stars (four for the selection process, two for comparability, and three for exposure/outcome), with a score of five or more indicating high quality. The results are presented in **Table 2**. Any discrepancies were resolved by consulting a consensus reviewer.

### Methods of Review

Relevant data from the included studies were extracted, critically appraised independently by two investigators using a structured sheet, and entered into a database. Any disagreements were resolved though discussions among the author group. The characteristics of the study and the patients were documented and are presented in a table format. We extracted operation time, intraoperative blood loss, the number of harvested lymph nodes, and the length of postoperative hospital stay to assess the effectiveness of surgical procedures and postoperative recovery. Postoperative complications, including anastomotic bleeding, leakage, stenosis, and reflux esophagitis, were compared. The rate of tumor recurrence and 5-year overall survival (OS) were used to estimate the safety of LPG and LTG. If necessary, the primary authors were contacted to retrieve further information.

### Statistical Analysis

Continuous variables, when both means and standard deviations (SDs) were presented, were assessed using the weighted mean difference (WMD) with 95% confidence intervals (CIs). If the study provided the median, range and size of a sample, we estimated the means and SDs according to published methods (28, 29). Dichotomous variables were calculated using the odds ratio (OR) and 95% CIs. Fixed-effects models were used for studies with low heterogeneity, while random effects models were used for those with high heterogeneity. Cochran’s Q-test was used to assess heterogeneity, and *P* < 0.1 was defined as significant. A sensitivity analysis was applied by removing individual studies from the data set and analyzing the effect on the overall results to identify sources of significant heterogeneity. Publication bias was evaluated using funnel plots and statistically tested by Begger’s test and Egger’s test. All statistical calculations were performed using Review Manager 5.3 (The Cochrane Collaboration, Oxford, UK) and STATA15.0 (Stata Corporation, College Station, TX, USA). A two-trailed value of *P* < 0.05 was considered statistically significant.

## Results

### Search Results

**Figure 1:**
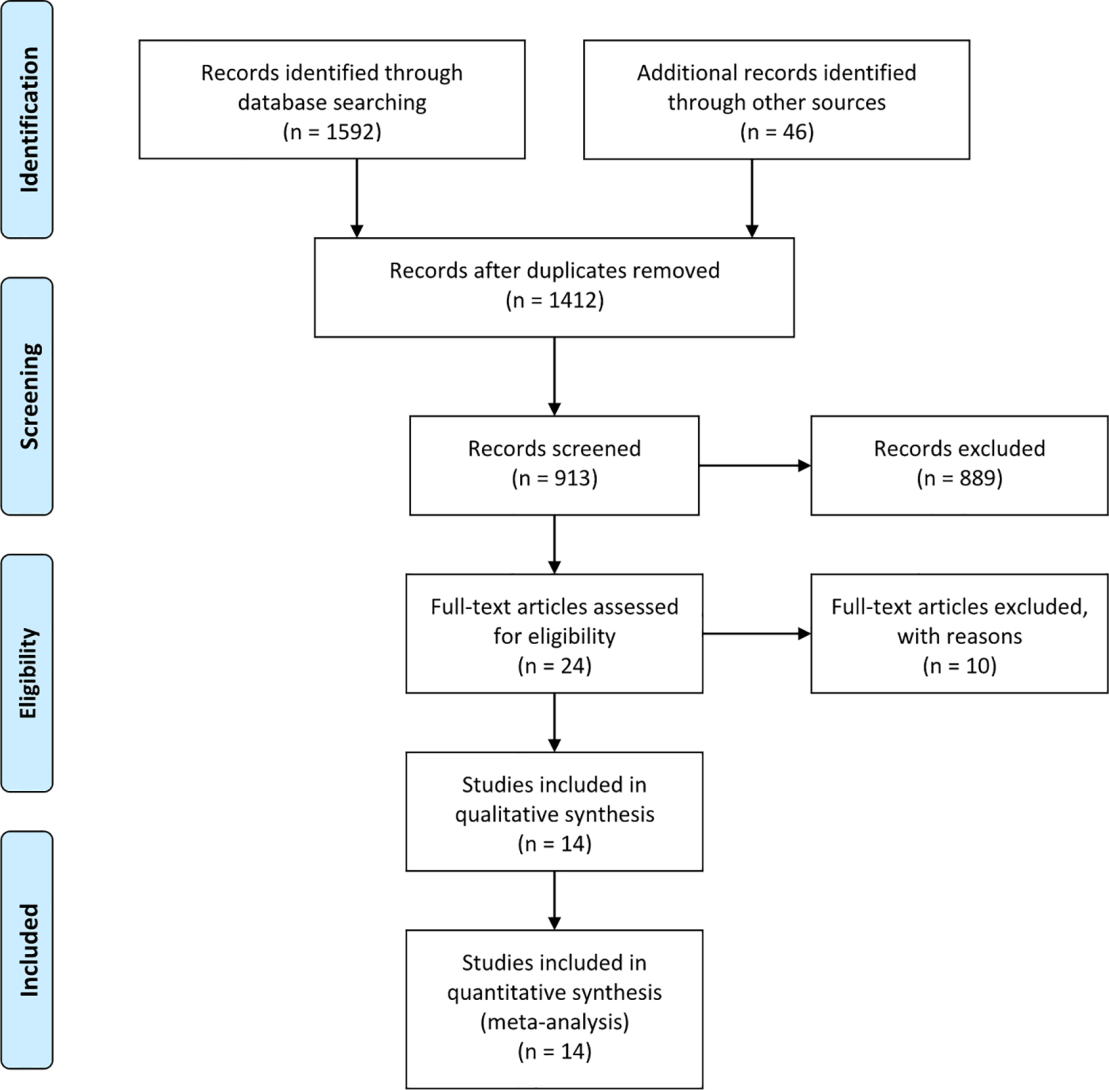
Articles identified with criteria for inclusion and exclusion.

The search strategy identified 1,632 articles that mentioned the use of LPG and LTG for proximal gastric cancer. After carefully screening the titles, abstracts, and full texts, 14 articles were selected based on the inclusion and exclusion criteria (30–43). **Figure 1** presents a flow diagram that details the selection process. All studies included were observational, and no randomized controlled studies were identified. The NOS was used to evaluate the quality of each study, and each study had a score of > 5 points.

### Characteristics of Included Studies

A total of 1,282 patients were involved in the meta-analysis (510 patients underwent LPG and 772 patients underwent LTG). The main characteristics of the fourteen studies included in the analysis are summarized in **Table 1**. All articles were published from 2007 to 2019; among these studies, nine trials were from Japan, four were from Korea, and one was from China.

**Table 1:** Characteristics of included studies.

Reference	Country	Approach	Sample size	Age (years)	Gender (M/F)	BMI (kg/m^2^)	Tumor size (cm)	TNM stage			Lymphadenectomy		Reconstruction	Follow-up duration (months)	NOS
								IA	IB	II	D1+	D2			
Tanimura et al. (30)	Japan	LTG	72	NR	NR	NR	NR	NR	NR	NR	NR	NR	R-Y	30 (3.6–85.2)	7
		LPG	38	NR	NR	NR	NR	NR	NR	NR	NR	NR	EG, JI		
Ahn et al. (31)	Korea	LTG	81	59.7 ± 11.8	56/25	23.6 ± 3.4	4.0 ± 2.7	NR	NR	NR	70	11	R-Y, EJ	38.3 ± 21.6	8
		LPG	50	58.8 ± 12.1	36/14	24.2 ± 3.7	2.8 ± 1.3	NR	NR	NR	50	0	EG	44.0 ± 17.9	
Kosuga et al. (32)	Japan	LTG	52	67 (40–89)	45/7	23.6 (19.0–42.8)	NR	45	7	0	50	2	R-Y, EJ	37.6 (3.5–71.3)	8
		LPG	25	66 (41–80)	17/8	22.3 (17.7–28.0)	NR	24	1	0	25	0	EG	36.1 (5.2–71.3)	
Kim and Kim (33)	Korea	LTG	17	60.9 + 12.9	10/7	23.4 + 5.0	4.0 + 3.2	12	3	2	NR	NR	EJ	NR	7
		LPG	17	64.7 ± 9.9	14/3	24.2 ± 3.8	2.2 ± 1.0	12	4	1	NR	NR	DT	NR	
Hosoda et al. (34)	Japan	LTG	59	66.5 ± 11.0	41/18	23.3 ± 3.5	5.1 ± 2.7	34	11	10	54	NR	EJ	42 (12–71)	9
		LPG	40	68.4 ± 8.3	32/8	23.5 ± 2.4	2.8 ± 1.2	32	4	3	29	NR	EG	37 (11–64)	
Jung et al. (35)	Korea	LTG	156	58.7 ± 10.8	120/36	23.9 ± 3.3	3.2 ± 1.9	NR	NR	NR	86	70	R-Y, EJ	43.5 ± 23.2	8
		LPG	92	59.8 ± 11.4	77/15	23.5 ± 2.7	2.4 ± 1.3	NR	NR	NR	92	0	DT	26.6 ± 10.3	
Nishigori et al. (36)	Japan	LTG	42	64.4 ± 12.2	28/14	22.8 ± 3.6	NR	NR	NR	NR	41	1	R-Y, EJ	50 (2–98)	9
		LPG	20	66.2 ± 13.4	15/5	23.4 ± 3.8	NR	NR	NR	NR	17	0	EG		
Hayami et al. (42)	Japan	LTG	47	69 (41–84)	34/13	22.4 (16.4–30.6)	34.5 (7–105)	42	2	0	47	NR	R-Y	49 (18–62)	8
		LPG	43	72 (37–90)	31/12	23.7 (18.2–36.2)	25.0 (8–70)	32	7	4	43	NR	DFT	25 (12–40)	
Park et al. (37)	Korea	LTG	46	56.7 ± 11.8	22/24	22.9 ± 3.4	3.2 ± 1.9	35	4	6	NR	NR	R-Y	47.5 (7.0–67.4)	8
		LPG	43	64.1 ± 12.2	26/8	23.1 ± 3.2	2.1 ± 1.1	29	1	3	NR	NR	DFT	29.6 (2.9–39.5)	
Sugiyama et al. (38)	Japan	LTG	20	68.6 ± 2.7	17/3	NR	NR	16	1	2	NR	NR	R-Y	12	7
		LPG	10	65.6 ± 3.8	7/3	NR	NR	8	1	1	NR	NR	DT	12	
Furukawa et al. (39)	Japan	LTG	48	63.5 (29–82)	35/13	22.2 (13.9–26.5)	32 (10–100)	39		4	32	16	R-Y	48.5	8
		LPG	27	70 (59–84)	22/5	22.8 (19.3–26.8)	25 (12–50)	24		2	27	0	DT	30	
Nomura et al. (40)	Japan	LTG	30	68.5 ± 8.3	21/9	NR	NR	17	4	8	NR	NR	R-Y	12	7
		LPG	30	67.5 ± 8.7	24/6	NR	NR	22	6	2	NR	NR	DT/JI	12	
Kano et al. (41)	Japan	LTG	78	66 (41–84)	66/12	NR	30 (1–75)	63	11	2	NR	NR	R-Y	60	9
		LPG	72	67 (30–88)	47/25	NR	28 (2–125)	61	6	3	NR	NR	JI/DFT	60	
Wang et al. (43)	China	LTG	24	58.38 ± 1.82	15/9	23.5 ± 0.69	2.12 ± 0.27	22		2	NR	NR	R-Y	12	7
		LPG	12	55.58 ± 4.10	6/6	23.29 ± 0.76	1.74 ± 0.17	10		2	NR	NR	DT	12	

LTG, laparoscopic total gastrectomy; LPG, laparoscopic proximal gastrectomy; BMI, body mass index; TNM, tumor, node, metastasis; NOS, Quality Assessment based on Newcastle-Ottawa Scale; R-Y, Roux-en-Y; EG, esophagogastrostomy; JI, jejunal interposition; EJ, esophagojejunostomy; DT, double tract anastomosis (gastrojejunostomy and jejunojejunostomy); DFT, double-flap technique; NR, not reported.

### Meta-Analysis Results

#### Surgery-Related Features

##### Operation Time

**Figure 2:**
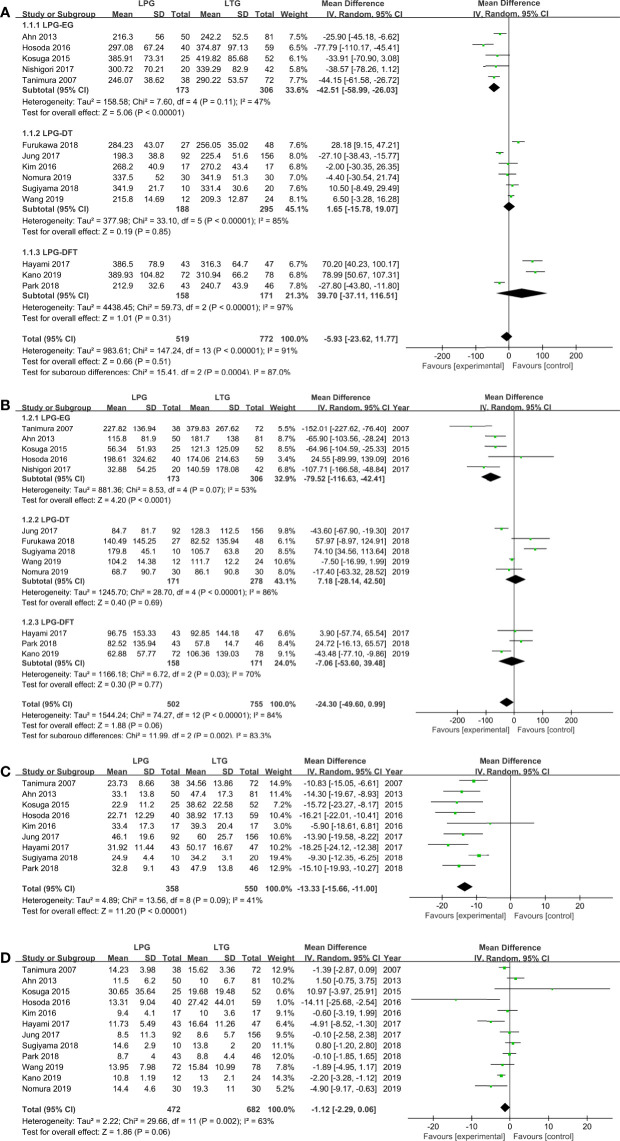
Meta-analysis comparing **(A)** operative time, **(B)** intraoperative blood loss, **(C)** harvested lymph nodes, **(D)** postoperative hospital stay.

All included studies (1,291 patients) provided data on operation time (30–43). No significant difference was found between these two groups (WMD = −5.92, 95% CI: −23.66 to 11.82, *P* = 0.51), and there was significant heterogeneity (*I* ^2^ = 91%, *P* < 0.00001) (**Figure 2A**, **Table 2**). In the subgroup analysis, no statistically significant differences were observed between the DT and DFT groups. However, the operative time was longer in the LTG group than in the LPG-EG group (WMD = −42.51, 95% CI: −58.99 to −26.03, *P* < 0.00001) (**Figure 2A**).

**Table 2:** Meta-analysis results of endpoints from all available studies.

Outcome of interest	No. of studies	No. of patients		Heterogeneity		Overall effect size	95% CI	*P* value
		LPG	LTG	*I* ^2^ (%)	*P* value			
Surgery-related features								
Operation time (min)	14	519	772	91	<0.00001	WMD = −5.92	−23.66 to 11.82	0.51
Blood loss (ml)	13	502	755	84	<0.00001	WMD = −24.30	−49.60 to 0.99	0.06
Harvested lymph nodes	9	358	550	41	0.09	WMD = −13.33	−15.66 to −11.00	< 0.00001
Postoperative hospital stay (day)	12	472	682	63	0.002	WMD = −1.12	−2.29 to −0.06	0.06
Postoperative complications								
Anastomotic leakage	10	329	515	0	0.83	OR = 1.13	0.56 to 2.28	0.74
Anastomotic bleeding	3	149	234	0	1.00	OR = 0.90	0.21 to 3.89	0.89
Anastomotic stenosis	10	354	552	24	0.23	OR = 2.03	1.21 to 3.39	0.007
Reflux esophagitis	9	344	532	53	0.03	OR = 1.87	0.74 to 4.71	0.18
Oncologic outcomes								
5-Year OS	5	274	416	39	0.16	OR = 1.04	0.47 to 2.27	0.93
Recurrent cancer	6	256	433	0	0.66	OR = 0.67	0.19 to 2.41	0.54

LPG, laparoscopic proximal gastrectomy; LTG, laparoscopic total gastrectomy; WMD, weighted mean difference; OR, odds ratio; OS, overall survival.

##### Blood Loss

Thirteen studies (1,257 patients) reported intraoperative blood loss volume (30–32, 34–43). No significant difference was observed between the two groups (WMD = −24.30, 95% CI: −49.60 to 0.99, *P* = 0.06), and the heterogeneity among the studies was significant (*I* ^2^ = 84%, *P* < 0.00001). In the subgroup analysis, the DT and DFT groups showed similar results except for the LPG-EG group (WMD = −79.52, 95% CI: −116.63 to −42.41, *P* < 0.0001) (**Figure 2B**).

##### Harvested Lymph Nodes

The quantities of harvested lymph nodes were included in nine studies (908 patients) and showed moderate heterogeneity (*I* ^2^ = 41%, *P* = 0.09) (30–35, 37, 38, 42, 43). The overall effect size favored the LTG group (WMD = −13.33, 95% CI: −15.66 to −11.00, *P* < 0.00001) (**Figure 2C**).

##### Postoperative Hospital Stay

Twelve studies (1,154 patients) provided data on postoperative hospital stay (30–35, 37, 38, 40–43). According to the random-effects model, there was no significant difference between the two groups (WMD = −1.12, 95% CI: −2.29 to −0.06, *P* = 0.06) (**Figure 2D**).

#### Postoperative Complications

**Figure 3:**
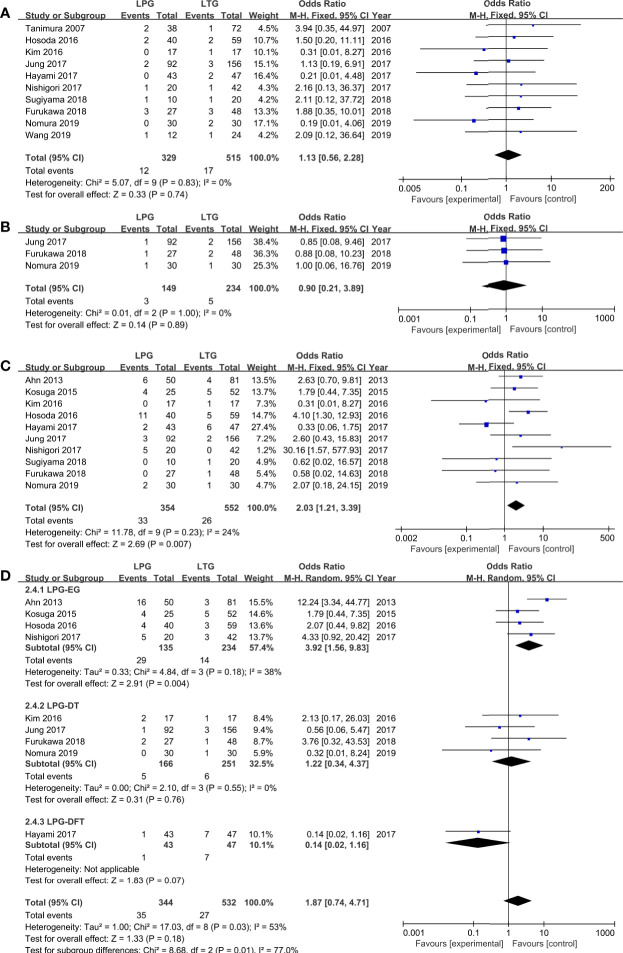
Meta-analysis comparing **(A)** anastomotic leakage, **(B)** anastomotic bleeding, **(C)** anastomotic stenosis, **(D)** reflux esophagitis.

Among the observed postoperative morbidities, there were no differences in the frequencies of anastomotic leakage and bleeding (**Figures 3A, B**, **Table 2**). The incidence of anastomotic stenosis was higher in the LPG group than in the LTG group (OR = 2.03, 95% CI: 1.21 to 3.39, *P* = 0.007, **Figure 3C**). Nine studies (876 patients) reported results for reflux esophagitis (31–36, 39, 40, 42). There was no significant difference between the two groups (OR = 1.87, 95% CI: 0.74 to 4.71, *P* = 0.18). The heterogeneity among the studies was moderate (*I* ^2^ = 53%, *P* = 0.03). In the subgroup analysis, compared to LPG-EG (OR = 3.92, 95% CI: 1.56 to 9.83, *P* = 0.004), the incidence of reflux between the LPG-DT and LTG groups was not significantly different (OR = 1.22, 95% CI: 0.34 to 4.37, *P* = 0.76, **Figure 3D**).

#### Oncologic Outcomes

##### Five-Year Overall Survival Rates

**Figure 4:**
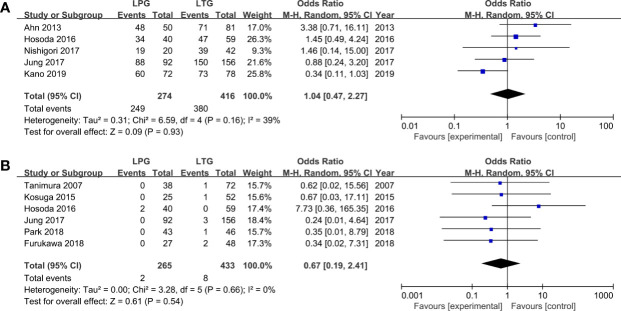
Meta-analysis comparing **(A)** 5-year OS, **(B)** recurrent cancer.

Five homogenous (*I* ^2^ = 39%, *P* = 0.16) studies (690 patients) reported 5-year overall survival rates (31, 34–36, 41). The results revealed that patients who had undergone LTG or LPG had similar 5-year overall survival rates (OR = 1.04, 95% CI: 0.47 to 2.27, *P* = 0.93, **Figure 4A**).

##### Recurrence Rate

Six studies reported data on recurrence and showed no heterogeneity (*I* ^2^ = 0%, *P* = 0.66) (30, 32, 34, 35, 37, 39). No statistically significant differences were observed between the LPG and LTG groups (OR: 0.67, 95% CI: 0.19 to 2.41 P = 0.54, **Figure 4B**).

### Sensitivity Analysis and Publication Bias

A sensitivity analysis was performed by excluding one study in turn to assess whether individual research influenced pooled ORs or WMDs. For every meta-analysis, the pooled ORs or WMDs were similar after each study was excluded, and this verified the stability of the meta-analysis.

**Figure 5:**
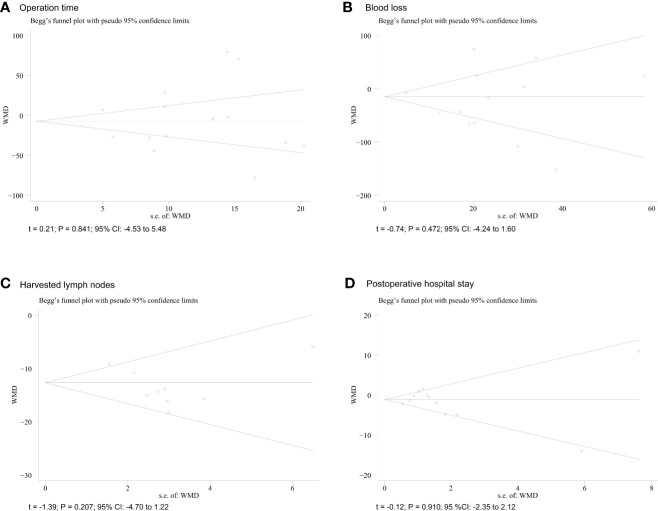
Funnel plots of each outcome. **(A)** operative time; **(B)** blood loss; **(C)** harvested lymph nodes; **(D)** postoperative hospital.

In our study, Egger’s tests were conducted to detect potential publication bias. The funnel plots for operative time, intraoperative blood loss, number of harvested lymph nodes, and length of postoperative hospital stay are shown for more than ten studies. According to the outcomes of shown in the funnel plot graphics, no indication of significant publication bias was observed (*P* > 0.05) (**Figure 5**).

## Discussion

The present meta-analysis assessed whether LPG is an acceptable alternative to LTG in patients with proximal gastric cancer. Our results suggest that compared to LTG, LPG resulted in fewer harvested lymph nodes. However, with regard for other parameters, there were no significant differences between the two methods. Based on the subgroup analysis of digestive tract reconstruction, however, classic LPG-EG had shorter operative time, less intraoperative blood loss and more reflux esophagitis. LPG-DT/DFT overcomes these shortcomings and has a similar prognosis. Recently, several meta-analyses have shown the superiority of LTG over LPG; however, these articles focused only on open surgery and did not evaluate laparoscopic surgery, and the use of different digestive tract reconstruction methods was not accounted for. With the development of laparoscopic techniques, the application of laparoscopic gastrectomy has been widely accepted, and several high-quality articles comparing LPG with LTG have recently been published. We therefore performed this meta-analysis to estimate the value of LPG in patients with proximal gastric cancer based on different reconstruction methods.

The results of this study show that the overall operative time and blood loss did not differ between LPG and LTG. There was a high degree of heterogeneity among the studies, and the results of the subgroup analyses suggest that this heterogeneity may be the result of differences in reconstruction methods. Traditional LPG-EG had shorter operative times and less blood loss; however, no statistically significant differences were observed between LPG-DT/DFT and LTG with regard for these parameters. The longer operative times and increased blood loss may be due to the complexity of the procedures performed in LTG and LPG-DT/DFT. During these procedures, abundant major nutritional vessels need to be identified, and numerous anatomic plans, complicated anastomosis methods, and extensive lymph node dissection need to be performed (44). Another explanation is that for surgeons performing these procedures, a complex learning curve is required to acquire proficiency. Cases performed by surgeons with less experience may take longer than subsequent cases performed by the same surgeon do due differences in skill (45, 46).

Because of its aggressive nature, proximal gastric cancer is usually diagnosed at a more advanced stage of the disease than is observed in distal gastric cancer (47). Thus, extended lymph node dissection and the number of harvested lymph nodes should be used to evaluate oncologic adequacy. The newly published Japanese Gastric Cancer Treatment Guidelines 2014 (ver. 4) recommend that proximal gastrectomy is only suitable for some early-stage diseases (44). Applying all aspects of the American Joint Committee on Cancer (AJCC) tumor, nodes, and metastasis (TNM) classification requires that the number of lymph nodes examined be at least 15. Based on our research, the mean number of lymph nodes retrieved by the two procedures was adequate in all included studies. In our analysis, we discovered that more lymph nodes were harvested during LTG than LPG, but no prognostic difference was observed between the groups. This result is in line with those presented in other similar studies (9, 12, 15, 19).

The incidence of postoperative complications is widely recognized as an important indicator of surgical safety. When considering the advantages of minimally invasive surgery and function-preserving procedures, LPG is theoretically a better option than LTG. However, most gastric surgeons are reluctant to perform proximal gastrectomy because of its two well-established complications: reflux and anastomotic stricture, or so-called anastomosis-related late complications (48, 49). Direct anastomosis between the esophagus and gastric remnant allows gastric acid to easily reflux to the esophagus, causing heartburn and regurgitation in some patients. Stricture may be due to the ischemia and inflammation caused by reflux at the anastomotic site, leading to fibrosis (12). In our analysis, we found that the incidence of anastomotic strictures was significantly higher in the LPG group than in the LTG group. In the subgroup analysis, the incidence rate of reflux esophagitis was also higher in the LPG-EG group (*P* = 0.004). These results corroborate the findings of many previous studies (12, 20, 21, 50). However, one of the more significant findings to emerge from this study is that LPG-DT has an incidence of reflux esophagitis similar to that of LTG. Hence, LPG-DT appears to be a safe, effective, and reliable reconstruction method with excellent postoperative outcomes in terms of preventing reflux symptoms.

The cancer recurrence and long-term survival rates are two visually effective outcomes for evaluating surgical interventions in oncological therapy. In this meta-analysis, we extracted 5-year overall survival (OS) from available articles. Based on our results, postoperative cancer recurrence and 5-year OS were similar between the LPG group and the LTG group, consistent with previous studies (9, 12).

Our study has novelty and multiple strengths. First, previous studies have focused only on open surgery and did not evaluate laparoscopic surgery. With the development of laparoscopic techniques, the application of laparoscopic gastrectomy has been widely accepted, and several high-quality articles comparing LPG with LTG have recently been published. We therefore performed this meta-analysis to estimate the efficacy and safety of the two procedures in patients with proximal gastric cancer. Second, we performed a subgroup analysis of patients with different reconstruction methods according to the subgroup analysis, different types of surgical procedure had different outcomes. To the best of our knowledge, this concern was largely absent from the current study. We believe that our findings can provide surgeons with valuable information when a minimally invasive surgical option is being considered.

There are several limitations that should be considered in this meta-analysis. First, the main body of literature in the current meta-analysis was observational, which may be mixed with some sources of bias. Second, all studies included were conducted in East Asian countries, potentially due to the high incidence of gastric cancer in eastern countries. The conclusions might therefore be biased toward Asian populations, and it may therefore be difficult to generalize these findings to other populations. Last, there was high heterogeneity in terms of operation time, blood loss and postoperative hospital stay. Differences in study design, sample size, and digestive tract reconstruction might explain this heterogeneity. Therefore, the addition of high-quality, multicenter, randomized, controlled trials from other countries and regions are needed to further clarify these issues.

## Conclusions

Because it produces fewer harvested lymph nodes and has a similar oncological safety profile, LPG can be performed as an alternative to LTG, especially LPG-DT/DFT, for proximal gastric cancer and has comparable safety and efficacy. Additional high-quality randomized controlled trials including Western patients and surgeons are still needed for further validation of these results.

## Data Availability Statement

The original contributions presented in the study are included in the article/supplementary materials, further inquiries can be directed to the corresponding author.

## Author Contributions

PT, PZ, and ZZ designed the study. SB, ML, MZ, and JL were involved in literature search and data interpretation. YL and LJ analyzed the data. PT wrote the manuscript. All authors contributed to the article and approved the submitted version.

## Funding

This work was supported in part by grants from the National Key Technologies R&D Program (No. 2015BAI13B09) and the National Key Technologies R&D Program of China (No. 2017YFC0110904).

## Conflict of Interest

The authors declare that the research was conducted in the absence of any commercial or financial relationships that could be construed as a potential conflict of interest.
